# Lead Poisoning among Opium Users in Iran: A Possible New Emerging Epidemic in the Region

**Published:** 2017-08

**Authors:** Seyed Ramin RADFAR, Pardis NEMATOLLAHI, Ali FARHOUDIAN, Alireza NOROOZI

**Affiliations:** 1. Substance Abuse and Dependence Research Center, University of Social Welfare and Rehabilitation Sciences, Tehran, Iran; 2. UCLA Integrated Substance Abuse Programs, Los Angeles, USA; 3. Dept. of Pathology, Isfahan University of Medical Sciences, Isfahan, Iran; 4. Dept. of Neuroscience and Addiction, School of Advanced Technologies in Medicine (SATiM), Tehran University of Medical Sciences, Tehran, Iran

## Dear Editor-in-Chief

Lead Poisoning (LP) is severe heavy metal intoxication with a wide range of symptoms such as high blood pressure, abdominal pain, constipation, joint pains, muscle pain, declines in mental functioning, pain, numbness or tingling of the extremities, headache, memory loss, mood disorders, reduced sperm count, abnormal sperm, miscarriage or premature birth in pregnant women (1) and anemia (2). LP could be fatal and there is an estimation that LP is responsible for 143000 deaths per year that mainly occurs in WHO South-East Asia Region, and WHO Western Pacific and Eastern Mediterranean Regions with 50% and 20% of total deaths, respectively (3).

On the other hand, from years ago there were reports regarding LP among opium users (4) and even methamphetamine users (5). In Iran, there were frequent reports of LP among opium users in less than decade ago (6–8). One of the possible causes for lead contamination opium is that the smuggler’s mix leads to increase the density of the opium (7), another possible hypothesis is soil pollution in Afghanistan with lead that needed to be investigated.

However, again after years of silence, there is evidence that shows a new epidemic of LP among opium users in Iran is rising. There are reports from a general hospital in a central city in Iran (Arak) that 18 cases of LP had been admitted to the hospital in Feb 2016. All were oral opium users and anemia was the main clinical manifestation among them (9). Another case that had been admitted to a referral hospital in Isfahan were a 49 yr old man with history of 10 yr of oral opium use, the main manifestation again were anemia with hemoglobin of 6.5 gr/dl, final diagnosis after bone marrow biopsy and serology tests were LP with blood level of 70 gr/dl.

Responsible authorities in Drug Control Head Quarter in Iran stated that most of the opium in black market is contaminated with lead and request drug users to stop opium using immediately and start their treatment in the clinics (10).

According to these data, until more findings and conduction of appropriated research, it is very important that healthcare providers not only those involved with drug abuse treatment. Moreover, all of the physicians especially general practitioners, internists and hematologists in Iran and other countries of the regions be aware of the high possibility of lead poisoning among their patients with history of drug use.
